# Efficacy of a T Cell-Biased Adenovirus Vector as a Zika Virus Vaccine

**DOI:** 10.1038/s41598-018-35755-z

**Published:** 2018-12-20

**Authors:** Brianna L. Bullard, Brigette N. Corder, Matthew J. Gorman, Michael S. Diamond, Eric A. Weaver

**Affiliations:** 1 School of Biological Sciences, Nebraska Center for Virology, University of Nebraska, Lincoln, USA; 2 Departments of Medicine, Molecular Microbiology, Pathology & Immunology, Washington University School of Medicine, Saint Louis, Missouri 63110 USA

## Abstract

Zika virus (ZIKV) is a major public health concern due to the risk of congenital Zika syndrome in developing fetuses and Guillain-Barre syndrome in adults. Currently, there are no approved vaccines available to protect against infection. Adenoviruses are safe and highly immunogenic vaccine vectors capable of inducing lasting humoral and cellular immune responses. Here, we developed two Adenovirus (Ad) vectored Zika virus vaccines by inserting a ZIKV prM-E gene expression cassette into human Ad types 4 (Ad4-prM-E) and 5 (Ad5-prM-E). Immune correlates indicate that Ad5-prM-E vaccination induces both an anti-ZIKV antibody and T-cell responses whereas Ad4-prM-E vaccination only induces a T-cell response. In a highly lethal challenge in an interferon α/β receptor knockout mice, 80% of Ad5 vaccinated animals and 33% of Ad4 vaccinated animals survived a lethal ZIKV challenge, whereas no animals in the sham vaccinated group survived. In an infection model utilizing immunocompetent C57BL/6 mice that were immunized and then treated with a blocking anti-IFNAR-1 antibody immediately before ZIKV challenge, 100% of Ad4-prM-E and Ad5-prM-E vaccinated mice survived. This indicates that Ad4-prM-E vaccination is protective without the development of detectable anti-ZIKV antibodies. The protection seen in these highly lethal mouse models demonstrate the efficacy of Ad vectored vaccines for use against ZIKV.

## Introduction

Zika virus (ZIKV) is a mosquito-borne, positive-stranded RNA virus that belongs to the genus Flavivirus in the *Flaviviridae* family^1^. This genus also contains other important human pathogens, such as Dengue, West Nile, Japanese encephalitis, and yellow fever viruses. ZIKV was first discovered in the Zika forest of Uganda in 1947 and believed to cause only asymptomatic or mild infection in humans^2^. However, the 2015 outbreak of ZIKV in Brazil showed higher than historical rates of congenital birth defects in fetuses and babies born to ZIKV-infected mothers along with an increase in the rate of Guillain-Barre syndrome in adults^3–6^. The World Health Organization (WHO) declared ZIKV a global public health emergency in February 2016 after an estimated 500,000–1,500,000 suspected cases of ZIKV infection with more than 4,300 cases of microcephaly were reported^7^. This state of emergency has since been lifted, although the importance of ZIKV research and vaccine development remains a global priority^8^.

Although there are no licensed vaccines available to prevent ZIKV infection, much progress has been made since 2015. Many vaccine platforms have been explored including traditional platforms such as live-attenuated^9–11^, inactivated^12–14^, and subunit vaccines^15–17^. Other platforms include the expression of ZIKV structural and non-structural genes in DNA^12,14,18–21^, mRNA^11,22–24^, or viral vectors^25–29^. The precursor-membrane (prM) and envelope (E) protein of ZIKV have been the primary structural antigens used in many recombinant vaccines and have shown promising results^11,12,14,18–28^. Importantly, it has been shown that expression of the prM and E ZIKV proteins leads to the assembly of ZIKV virus-like particles (VLPs) *in vitro*^30^, which could be beneficial in a gene-delivery based vaccine platform, as VLPs can induce highly neutralizing antibodies that recognize conformational epitopes on the viral envelope (E) protein.

Adenovirus viral vectors have been shown to induce both strong humoral and cellular immunity^31,32^. Adenovirus type 5 (Ad5) has been the most extensively used vector due to the robust immune response it elicits. However, pre-existing immunity to Ad5 has been reported to be as high as 82.5% in Brazil and 70% in the United States^33^. With high pre-existing immunity to Ad5, other Adenovirus types have been explored. A epidemiological study of military recruits in the United States, showed that only 33% had pre-existing immunity to human Adenovirus type 4 (Ad4)^34^. Ad4 has been shown to be effective as a viral vector in vaccination against influenza, either alone or in combination with Ad5^35,36^.

In this study, we developed both human Ad5 and Ad4 expressing the ZIKV full-length prM-E genes as a candidate ZIKV vaccine. Ad5-prM-E elicited both a strong antibody and T-cell response, whereas Ad4-prM-E did not induce any detectable anti-ZIKV antibodies but still induced a strong T-cell response. In a lethal ZIKV challenge model using an *Ifnar*^*−/−*^ mice, Ad5-prM-E provided superior protection to Ad4-prM-E vaccination. However, both Ad vectors protected 100% of mice in a challenge model using anti-Ifnar1 blocking antibody. These data support the use of Ad vectors as a platform for ZIKV vaccine development.

## Results

### Construction of replication-defective Adenoviral-vectored vaccines

**Figure 1:**
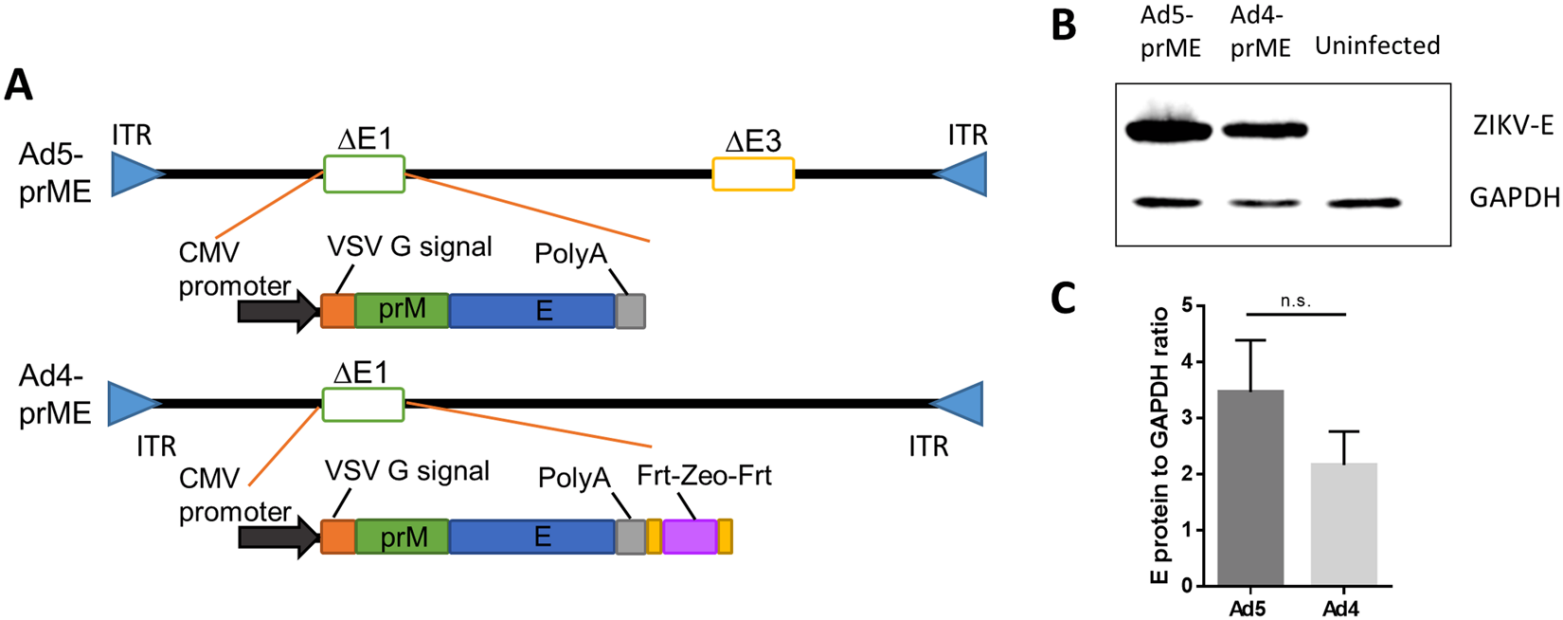
Construction and characterization of Adenovirus-vectored vaccine. (**A**) The ZIKV strain PRVABC59 prM-E region with a VSV G signal peptide under the control of a CMV promoter was cloned into the Ad5 and Ad4 genomes in the E1 region. Ad5 was also deleted for the E3 region. (**B**) A representative western blot comparing the levels of ZIKV-E protein expression from Ad4-prM-E and Ad5-prM-E infected 293 cells infected at an MOI of 1. GAPDH is used as a cellular control. (**C**) The ratio of ZIKV-E protein to GAPDH of western blots from C. The western blot data represents three separate biological replicates (n.s. not significant, two-tailed t test). Data are expressed as the mean with standard error (SEM).

The full length prM-E genes of Zika virus (ZIKV) strain PRVABC59 (Puerto Rico, 2015) were cloned into the E1 region of Ad type 5 (Ad5-prM-E) or Ad type 4 (Ad4-prM-E) to create a replication-defective vector (Fig. 1A). The Ad5-prM-E vector is also E3 deleted to increase cloning capacity. In addition, the Zeocin resistance gene flanked by Frt regions (which was used for effective screening of recombinant clone) was left in the Ad4 vector for simplicity. However, this gene can be easily removed using FLP recombinase for future studies. These differences should not affect *in vivo* immunogenicity as both of these vectors are replication defective. ZIKV-E protein expression was confirmed in both Ad vaccines by western blotting (Fig. 1B). 293 cells were infected at on MOI of 1 for 48 hours and cell lysate was collected. No significant difference in ZIKV-E protein expression was detected. Data was collected from three separate biological replicates (Fig. 1C).

### Antibody response to vaccination

**Figure 2:**
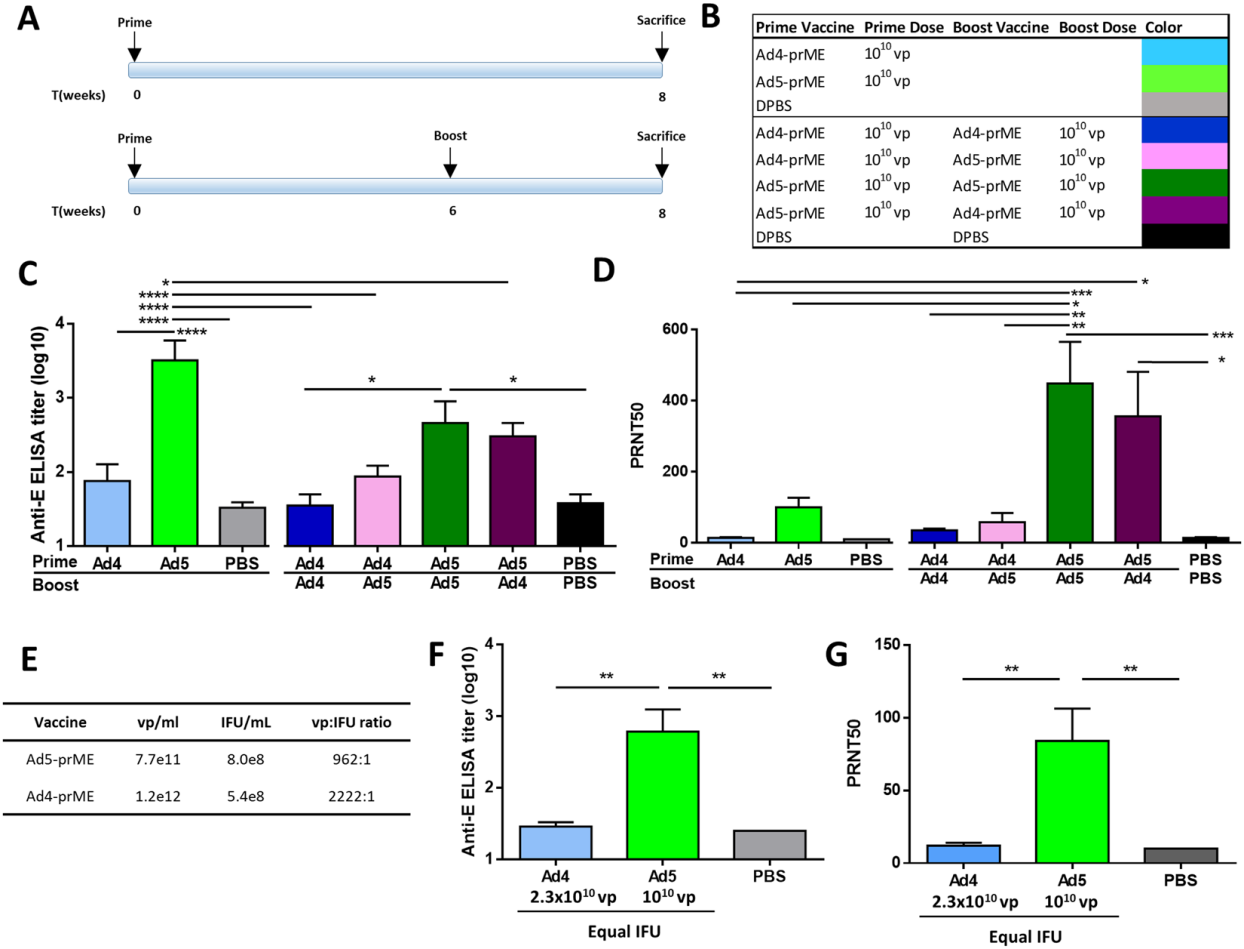
Antibody response from Adenovirus vaccination. (**A–D**) C57BL/6 mice (n = 5) were immunized with either Ad5-prM-E, Ad4-prM-E, or sham vaccine according to the timeline in (**A**) and at the indicated dose via the i.m. route (**B**). ZIKV E protein specific antibodies were measured using an ELISA in (**C**) and neutralization titer (PRNT50) was determined by plaque reduction assay in (**D**) (^*^p < 0.05; ^**^p < 0.01, ^***^p < 0.001, ^****^p < 0.0001; one-way ANOVA with Bonferroni multiple comparisons). To ensure that the undetectable antibody response to Ad4-prM-E vaccination was not sue to differences in infectivity of Ad vaccine virus stocks, the virus particle (vp) to infectious unit (IFU) ratio was determined (**E**). C57BL/6 mice (n = 5) were then vaccinated with an equal IFU dose and the antibody response measured via ELISA (**F**) and plaque reduction assay (**G**) ^**^p < 0.01; one-way ANOVA with Bonferroni multiple comparisons). Data are expressed as the mean with standard error (SEM).

To examine the humoral immunity induced by vaccination, groups of C57BL/6 mice were immunized with a single dose of 10^10^ virus particles (vp) of Ad vaccine (Ad5-prM-E or Ad4-prM-E) by the intramuscular route (i.m.) on day 0 (Fig. 2A,B). PBS was used as a vaccination negative control. All vaccinated mice were sacrificed on week 8 for immune response analysis. Mice that received single priming dose of Ad5-prM-E showed significantly higher levels of anti-ZIKV E protein antibodies as measured by ELISA (Fig. 2C) compared to the control PBS-treated mice. However, Ad4-prM-E vaccinated mice did not show detectable levels of specific anti-ZIKV-E protein antibodies or neutralizing antibodies (Fig. 2C,D).

To investigate if an Ad prime/boost vaccination strategy would increase immune responses, C57BL/6 mice were vaccinated at day 0 with 10^10^ vp of Ad vaccine, boosted with 10^10^ vp of the homologous or heterologous Ad vaccine on week 6, and sacrificed on week 8 (Fig. 2A,B). Prime/boost strategies utilizing Ad5-prM-E as the prime showed significantly higher levels of neutralizing antibodies than control mice (Fig. 2C,D). In contrast, the Ad4-prM-E primed mice showed no boosting effect at all.

### Ad4-prM-E vaccination does not induce detectable antibodies

To ensure that the lack of antibody response during Ad4-prM-E vaccination was not due to infectious differences in the Ad vaccine virus stocks, C57BL/6 mice were vaccinated based on infectious units (IFU). The IFU per mL was calculated for both Ad5-prM-E and Ad4-prM-E vaccine stocks. An analysis of virus particle (vp) to IFU showed a 2.3-fold difference between Ad5-prM-E and Ad4-prM-E (Fig. 2E). Mice were then vaccinated with 10^10^ vp of Ad5-prM-E or 2.3 × 10^10^ vp of Ad4-prM-E, resulting in a vaccination dose with equal IFU. In order to evaluate the response to vaccination near the predicated peak immune response, mice were euthanized 2 weeks later for immune correlate analysis. Again, Ad4-prM-E vaccination did not result in any detectable anti-ZIKV antibodies as measured by ELISA (Fig. 2F) or plaque reduction neutralization test (Fig. 2G). This data indicates that the lack of antibody development in Ad4-prM-E vaccinated mice is not due to differences in vaccine stock infectivity.

### T-cell responses after vaccination

**Figure 3:**
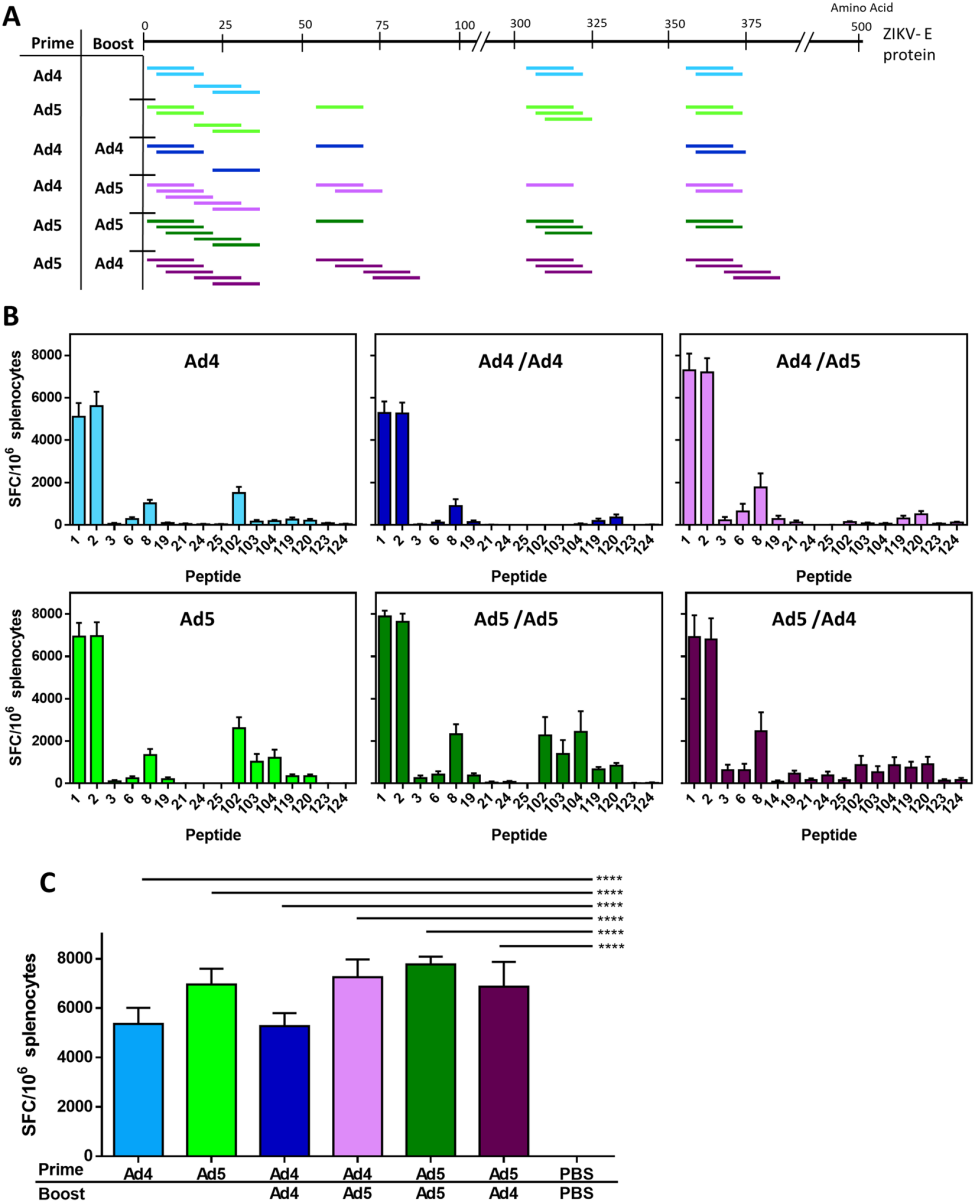
T-cell epitope mapping in response to vaccination. (**A**,**B**) Splenocytes from the vaccinated C57BL/6 mice (n = 5) were harvested. T-cell epitopes against ZIKV-E protein were mapped using a 164 peptide array of 15-mers with 13 amino acid overlap spanning the entire ZIKV-E protein. Peptides were considered positive if the response was 4 times the sham vaccinated mice. Positive peptides for each vaccination group are indicated in (**A**). The level of response seen against each positive peptide are reported as spot-forming cells (SFC) per million splenocytes (**B**). Peptides 1 and 2 encompass a previously described CD8^+^ immunodominant T-cell epitope^37^. The T-cell response against this epitope is shown as spot-forming cells (SFC) per million (**C**) (^****^p < 0.0001; one-way ANOVA with Bonferroni multiple comparisons). Data are expressed as the mean with standard error (SEM).

The T-cell response against the tested vaccine strategies and ZIKV infection was examined using an IFN-γ enzyme-linked immunospot assay (ELISPOT). Splenocytes were isolated and incubated with 15-mer peptides with 13 amino acid overlap spanning the entire E protein of ZIKV strain PRVABC59. Of the 164 tested peptides, 16 peptides were positive for a response (Fig. 3; Table. S1). A response was considered positive if it was 4 times the response of the PBS sham vaccinated mice (Fig. 3A). Both Ad5-prM-E and Ad4-prM-E vaccination induced a significant T-cell response against ZIKV E protein peptides (Fig. 3B). Peptide 1 and 2 induced the strongest T-cell response in all Adenovirus vaccinated animals (Fig. 3C) and encompassed the previously identified immunodominant CD8^+^ T-cell epitope E_4–12_ (position number E_294–302_ on the polypeptide)^37,38^. A strong T-cell response was generated against this epitope after a single shot of either Ad4-prM-E or Ad5-prM-E and was similar in intensity to the T-cell response seen after being boosted with either the homologous or heterologous Ad vaccine. Therefore, the prime/boost strategy and Adenovirus serotype-switching strategy did not significantly increase the T cell responses as judged by IFN-γ ELISPOT.

### Vaccine protection to lethal ZIKV infection in mice

**Figure 4:**
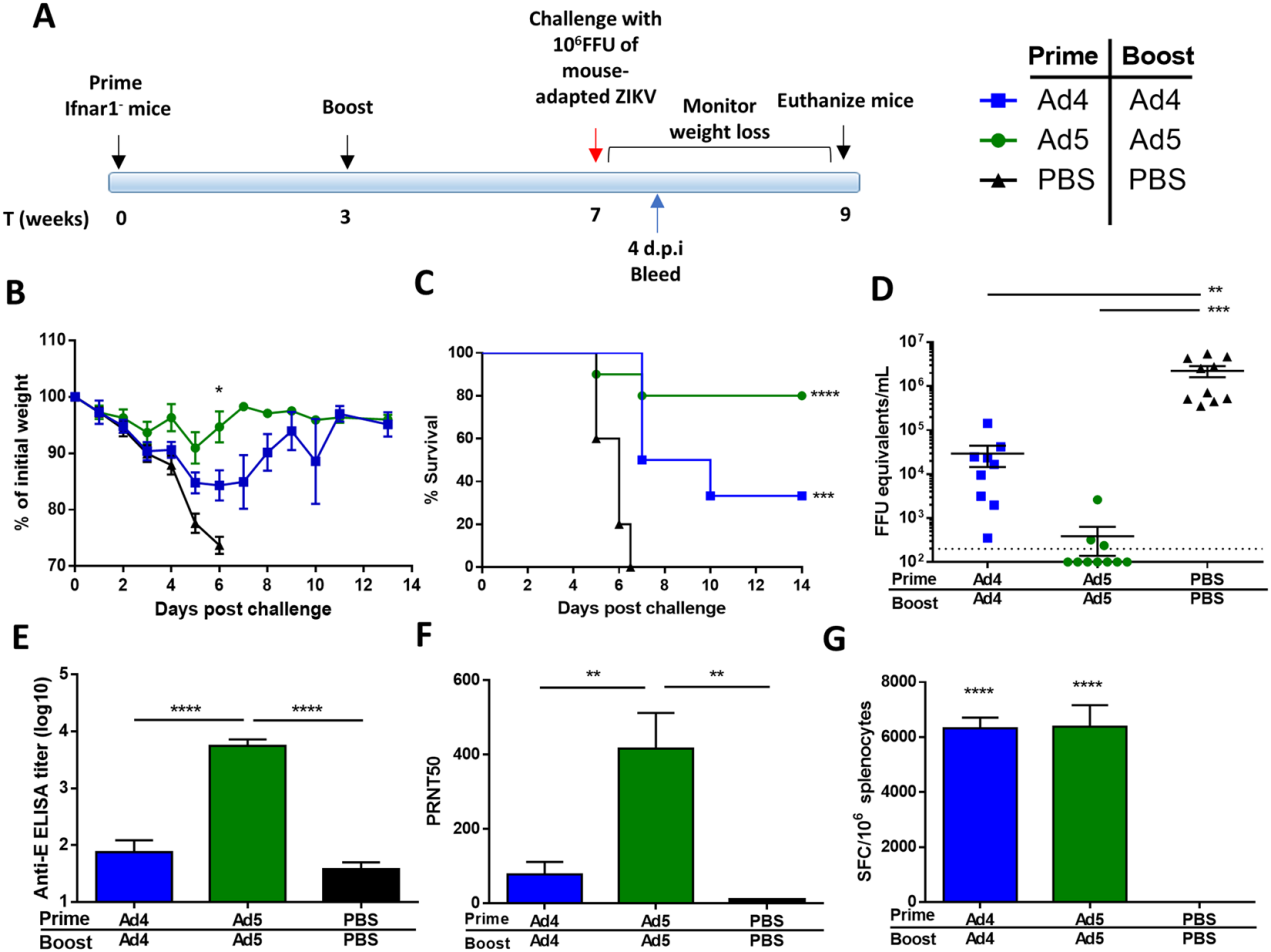
Vaccine protection against challenge in *Ifnar1*^*−/−*^ mice. (**A–D**) *Ifnar1*^*−*/*−*^ mice were immunized with 10^10^ vp of Ad4-prM-E (n = 9), Ad5-prM-E (n = 10), or sham PBS (n = 10) via the i.m. route according to the timeline in (**A**). Two experimental replicates were performed. Mice were boosted with the same vaccine and dose at week 3 and then challenged with 10^6^ FFU of mouse-adapted ZIKV strain 41519 at week 7. Weight loss was monitored (**B**) and mice were sacrificed when a 25% weight loss was reached (**C**). Asterisks indicate significance in weight compared to PBS sham vaccinated mice as determined by two-way ANOVA (p < 0.05). Survival data was analyzed using a log rank test (^***^p < 0.001, ^****^p < 0.0001). Blood was sampled at 4 days post infection (d.p.i) to determine the viral load in the sera using RT-qPCR (**D**) (^**^p < 0.01; ^****^p < 0.0001; one-way ANOVA). To determine immune correlates in *Ifnar1*^*−*/*−*^ mice, groups (n = 5) were immunized with 10^10^ vp of the indicated Ad vaccine and sacrificed 2 weeks later. Sera was used to determine anti-ZIKV IgG (**E**) and neutralizing antibodies (**F**). In addition, an ELISPOT was performed to determine spot-forming cells per million splenocytes against the immunodominant E_4–12_ epitope (**G**). ELISA, PRNT50, and ELISPOT are analyzed with one-way ANOVA (^**^p < 0.01, ^****^p < 0.0001). Data are expressed as the mean with standard error (SEM).

Initially, protection was examined in *Ifnar1*^*−*/*−*^mice on a C57BL/6 background, which are vulnerable to ZIKV infection^39^. Since no significant differences were seen in immune correlates between vaccination based on virus particles or infectious units (Fig. 2), challenge studies were carried out with 10^10^ vp per mouse. *Ifnar1*^*−*/*−*^ mice were primed via i.m. injection at day 0 with 10^10^ vp Ad4-prM-E or Ad5-prM-E, or PBS as a sham control. Animals were boosted with the same dose of vaccine at week 3 and challenged at week 7 with 10^6^ FFU of mouse-adapted ZIKV strain 41525^40^ (Fig. 4A). A comparison of the genetic differences between the PRVABC59 vaccination strain and the Dakar 41525 challenge strain can be seen in Supplementary Fig. 1. Studies have identified a single ZIKV serotype indicating that one ZIKV vaccine antigen should protect against all ZIKV strains^13,41^. After challenge, mice were monitored and euthanized when a weight loss of 25% or more was detected. Ad5-prM-E primed and boosted mice showed an 80% survival rate and Ad4-prM-E primed/boost mice had a 33% survival rate whereas all of the sham vaccination animals succumbed by day 7 (Fig. 4B,C; Fig. S2A). ZIKV viremia in mice peaks between day 3–4 post^42,43^, so animals were bled 4 days after infection to determine viremia using RT-qPCR (Fig. 4D). Ad5-prM-E animals showed a highly significant decrease in viremia (p < 0.001). Ad4-prM-E vaccination also decreased viremia compared to those in sham vaccinated mice. In addition, a plaque assay was performed to determine infectious ZIKV levels in sera. Both Ad4-prM-E and Ad5-prM-E vaccination significantly reduced infectious plaque forming units in the sera (Fig. S2B**)**. To ensure that the response to vaccination in this immunocompromised mouse is intact, immune correlate analyses were performed after priming and boosting of *Ifnar1*^*−*/*−*^ mice. ELISA (Fig. 4E), PRNT50 (Fig. 4F), and ELISPOT (Fig. 4G) confirmed a vaccine-induced immune response that was similar to wild type C57BL/6 mice.

**Figure 5:**
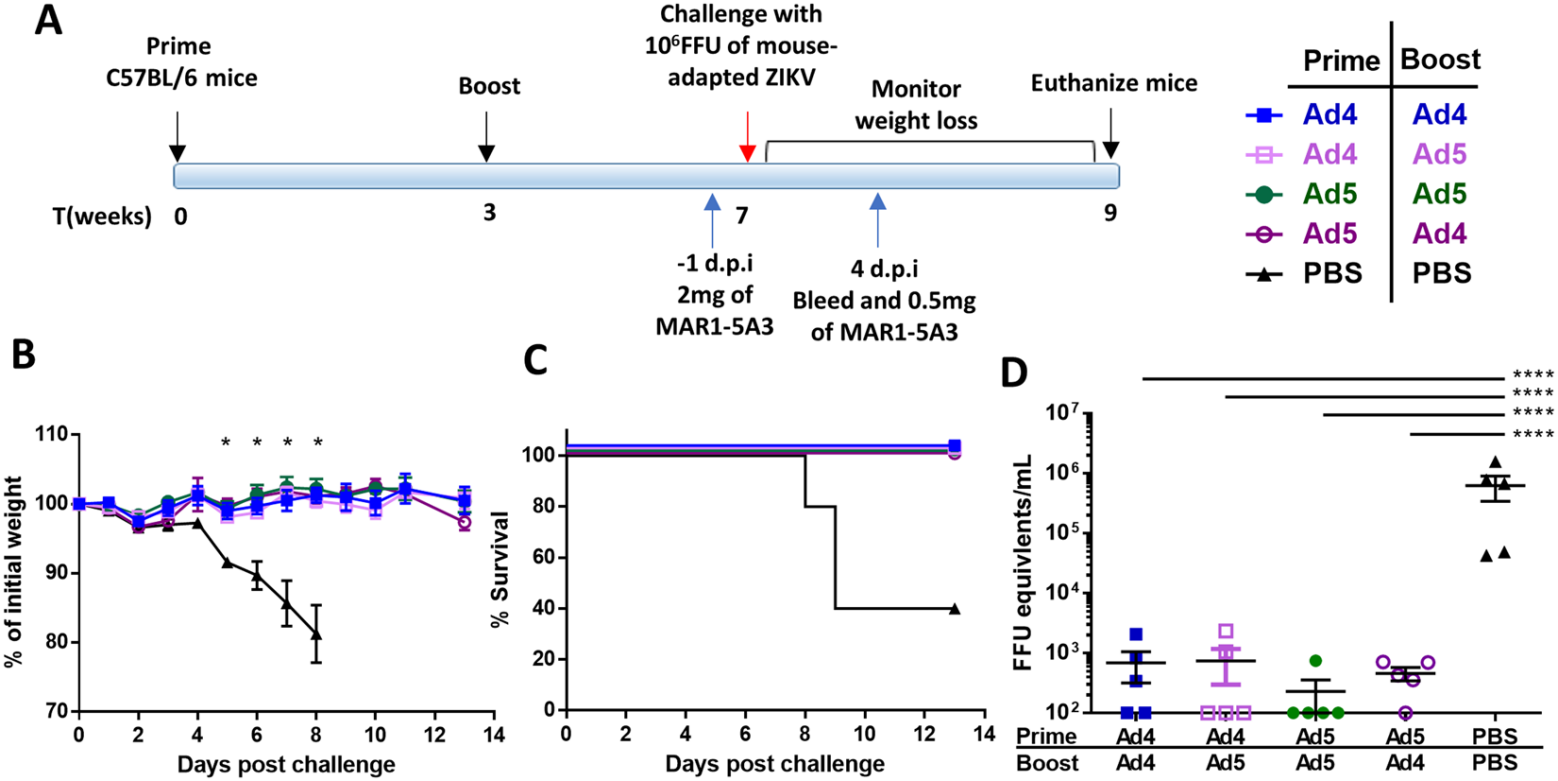
Vaccine protection against challenge in C57BL/6 mice in anti-Ifnar1 mAb-treated mice. (**A–D**) C57BL/6 mice (n = 5) were immunized with 10^10^ vp of Adenovirus vectored vaccine or sham PBS via i.m. injection according to the timeline in (**A**). Mice were boosted with at week 3 with the indicated vaccine. At week 7, mice were challenged with 10^6^ FFU of mouse-adapted ZIKV strain 41525. One day prior to infection, 2 mg of mouse anti-Ifnar1 was administered via i.p. injection to create a lethal challenge model. Another 0.5 mg of antibody was administered on 4 days post infection (d.p.i). Weight loss was monitored (**B**) and mice were sacrificed at 25% weight loss (**C**). Asterisks indicate significant in weight compared to PBS sham vaccinated mice as determined by two-way ANOVA (p < 0.05). Blood was sampled at 4 d.p.i to determine the viral load in the sera using qPCR (**D**) (^****^p < 0.0001; one-way ANOVA). Data are expressed as the mean with standard error (SEM).

Due to the immunocompromised nature of the *Ifnar1*^*−*/*−*^ mice, we tested a second challenge model that utilized wild type C57BL/6 mice and a blocking anti-Ifnar1 antibody (MAR1-5A3) administered after vaccination but before challenge, which makes naïve mice susceptible to lethal ZIKV infection^43^. Groups of C57BL/6 mice were immunized via the i.m. route with 10^10^ vp of Ad4-prM-E or Ad5-prM-E, or PBS (Fig. 5A). Mice were boosted at week 3 with 10^10^ vp of Adenoviral vector, either heterologous or homologous to the prime Adenovirus type. One day prior to challenge, mice were administered 2 mg of anti-Ifnar1 mAb via i.p. injection. An additional 0.5 mg of mAb was administered on day 4 post infection to ensure lethal challenge. At week 7, all mice were challenged with 10^6^ FFU of mouse adapted ZIKV Dakar strain 41525 and monitored for weight loss (Fig. 5B). All Adenoviral vaccinated mice survived challenge, regardless of prime/boost strategy (Fig. 5C). In comparison, all sham vaccinated mice showed significant weight loss by day 5 (Fig. 5B). All Adenovirus vaccinated animals showed decreased viremia compared to sham vaccinated mice by RT-qPCR (Fig. 5D) and plaque assay (Fig. S2C).

## Discussion

Adenoviruses have been widely used as vaccine vectors due to their ability to induce humoral and cellular immune responses^44–46^. Replication-defective Adenovirus vectors have been shown to be safe and well tolerated in clinical trials^32,47,48^. Here we developed replication-defective human Ad5 and Ad4 vectored ZIKV vaccines and characterized their immune responses and protection against ZIKV infection. Ad5-prM-E vaccination induced a strong anti-ZIKV humoral immune response. However, Ad4-prM-E vaccination induced no detectable anti-ZIKV antibodies, even with vaccination with an equal IFU dose to the Ad5-prM-E vaccination or in a prime-boost strategy.

Vaccination by either Ad5-prM-E and Ad4-prM-E resulted in the development of a T-cell immune response. This is important because CD8^+^ T-cells have been shown to protect against ZIKV infection^37,38,49^. Notably, both Ad vectored vaccines elicited T-cell responses against the immunodominant epitope E_4–12_. The ability of Ad4-prME vaccination to induce significant T-cell response but no detectable antibody response could demonstrate preferential development of cellular over humoral immune response in Ad4 vectored vaccination. Ad has a self adjuvanting property, and the Ad4 and Ad5 vectors may initiate very different cytokine profiles that are highly Th_1_ biased in Ad4 immunized mice but more Th_1_/Th_2_ balanced in the Ad5 immunized mice^50^. A phase I clinical trial utilizing Ad4 as an oral, replication-competent vector for H5 influenza vaccination found similar results. Participants vaccinated with an Ad4-H5 vector developed robust T-cell responses but failed to seroconvert even after 3 vaccinations (11% in vaccinated vs 7% in placebo groups)^51^. This could indicate that the immune profile of a strong T cell response with limited antibody development that we see with our Ad4-prM-E vaccination is not limited to mouse models or the ZIKV antigen, but further studies are needed to fully evaluate this.

In the anti-Ifnar1 blockade challenge model, both Ad5-prM-E and Ad4-prM-E vaccinated mice protected against weight loss and death. On day 4 post infection, vaccination significantly reduced levels of ZIKV RNA and infectious virus in serum. Even in the highly lethal *Ifnar1*^*−*/*−*^ mouse challenge model, the Ad vectored vaccines were partially protective. The immunocompromised state of the mice along with the challenge dose made this challenge highly stringent. However, as ZIKV infection is not lethal in adult human infection, further studies need to be performed to determine if the partial protection in this highly lethal challenge study translates to protective efficacy in human vaccination.

In conclusion, we constructed two replication-defective Adenovirus (Ad) vectored vaccine candidates by inserting the ZIKV structural prM-E genes into the E1 region of human Ad type 4 and Ad type 5. Both vectors have shown promise by protecting against ZIKV infection in a lethal antibody blockade challenge model. However, immune correlates indicate that Ad4-prM-E vaccination established a different immune profile than Ad5-prM-E vaccination. The ability of Ad4-prM-E vaccination to induce a strong T-cell response with undetectable levels of antibody, while Ad5-prM-E vaccination induces both strong antibody and T-cell responses, warrants further investigation. A comprehensive cytokine/chemokine and cellular profile analysis may elucidate these mechanisms. Due to the potential for antibody dependent enhancement (ADE) of Dengue virus from ZIKV antibodies^22,52,53^, the lack of antibody development in Ad4-prM-E vaccination could be explored as a potentially safer vaccine platform. Our study highlights the significant differences in immune profiles between these two Ad vectors and supports further investigation of the use of Ad vectors as a platform for ZIKV vaccine development.

## Materials and Methods

### Ethics statement

B6(Cg)-Ifnar1^tm1.2Ees^/J *Ifnar1*^*−*/*−*^ mice were purchased from Jackson Laboratories and a breeding colony was established. Female C57BL/6 J mice ages 6–8 weeks were purchased from Jackson Laboratory. Mice were housed in Life Sciences Annex building on the University of Nebraska – Lincoln (UNL) campus under the Association for Assessment and Accreditation of Laboratory Animal Care International (AAALAC) guidelines. The protocols were approved by the UNL Institutional Animal Care and Use Committee (IACUC) (Project ID 1448: Viral Vectored Flavivirus Vaccines). All animal experiments were carried out according to the provisions of the Animal Welfare Act, PHS Animal Welfare Policy, the principles of the NIH Guide for the Care and Use of Laboratory Animals, and the policies and procedures of UNL. All immunizations and bleeds were performed under ketamine and xylazine induced anesthesia.

### Viruses

Zika virus (ZIKV) strain PRVABC59 (ZIKV-PR; Puerto Rico, 2015) was provided by the Biodefense and Emerging Infections Research Resources Repository (BEI Resources). ZIKV strain Dakar 41525 was adapted for mice by passage in Rag1^*−*/*−*^ mice^40^. Viral stocks of ZIKV strain PRVABC59 were grown on C6/36 cells and collected after 7 days. Viral stocks of mouse-adapted ZIKV strain Dakar 41525 were grown on Vero cells and harvested after 3 days. Supernatant was collected and clarified by centrifugation at 350 g for 10 min. Aliquots were stored at −80 °C. The virus was quantified by plaque assay or focus forming assay (FFA)^39^.

### Recombinant adenovirus type 5 plasmid construction

The full prM-E gene from ZIKV strain PRVABC59 was codon-optimized for human gene expression, and the VSV G signal sequence was added to the N-terminus of the protein. The DNA was synthesized by GenScript and kindly provided by Dr. Asit Pattnaik and cloned in a pCI-Neo mammalian expression vector. Recombinant Adenovirus 5 was created using the AdEasy Adenoviral Vector System (Agilent). Briefly, to introduce unique restriction enzyme sites for downstream cloning, the prM-E region was amplified by PCR from the pCI-Neo DNA plasmid vaccine using the Q5 High-Fidelity 2X Master Mix (NEB). It was cloned into pCR-Blunt using the Zero Blunt TOPO PCR Cloning Kit (Invitrogen) for sequence confirmation. After confirmation, the gene was cloned into the pShuttle-CMV supplied in the AdEasy kit using T4 DNA ligase (NEB). This shuttle contains both a CMV promoter and a SV40 PolyA signal. The pShuttle-CMV-prM-E was linearized and transformed into BJ5183 electrocompetent cells with the pAdEasy-1 vector (Adenovirus type 5) to undergo homologous recombination. This recombination inserts the prM-E expression cassette into the E1 region of the Adenovirus type 5 (Ad5) genome. In addition, this vector is also deleted for the E3 region. Recombination was confirmed by restriction digest and a successful recombinant was transformed into XL1 cells for midiprep using the Qiagen Plasmid Midi Kit (Qiagen).

### Recombinant adenovirus type 4 plasmid construction

The Adenovirus type 4 genome was cloned and a shuttle plasmid to replace the E1 region was created as previously described^35^. Briefly, the complete Adenovirus type 4 (pAd4) genome was cloned into a single low-copy plasmid, and a shuttle plasmid (pAd4-recomb-cloner) to replace the E1 region of Ad4 was created using overlapping PCR products. The CMV-prM-E-PolyA region from the pShuttle-CMV-prM-E plasmid was amplified using the Platinum PCR SuperMix High Fidelity (Invitrogen) and fused to an Frt-Zeo-Frt fragment using overlapping PCR. This fragment was then cloned into pCR8/Gw/TOPO for sequence confirmation (Invitrogen). After sequence confirmation, the shuttle was linearized and transformed with the pAd4 plasmid into BJ5183 electrocompetent cells for homologous recombination. Recombinants were screened on kanamycin and zeocin and confirmed with restriction digest. A successful recombinant was then transformed into XL1 cells, and plasmid DNA was purified using the Qiagen Plasmid Midi Kit (Qiagen).

### Recombinant adenovirus virus rescue and purification

The recombinant Adenovirus 5 and 4 genomes with the ZIKV prM-E insert were linearized and buffer exchanged using a Strataprep PCR purification kit (Agilent Technologies). The linearized recombinant gDNA was transfected into 293 cells using the PolyFect Transfection Reagent (Qiagen). After virus rescue was observed via plaque formation, cells were harvested and virus was released by 3 freeze-thaw cycles. Virus was amplified by sequential passages in 293 cells until a final amplification using a Corning 10-cell stack (~6300 cm2). The virus was purified by 2 sequential CsCl ultracentrifuge gradients, desalted using Econo-Pac 10DG Desalting Columns (Bio-Rad), and stored at −80 °C in Ad-tris buffer (20 mM Tris-HCl, 100 mM NaCl_2_, 1 mM MgCl_2_•6H_2_O, 10% glycerol).

### Recombinant adenovirus quantification

After CsCl purification and desalting, the virus particle quantity was determined on a NanoDrop Lite spectrophotometer (Thermo Fisher) with an OD260. The infectious unit titer was determined using the QuickTiter Adenovirus Titer Immunoassay Kit (Cell Biolabs, Inc.). Briefly, 293 cells were infected with serial dilutions of Adenovirus stock in triplicate. After incubation for 48 hours, cells were fixed with cold methanol, blocked with PBS with 1% BSA, and then incubated with anti-hexon antibody. Cell were washed, incubated with secondary HRP-conjugated antibody, and developed with DAB. Positive cells were stained brown and counted to determine the infectious units per mL.

### Western blotting

For confirmation of protein expression from the recombinant Adenoviruses, confluent 293 cells were infected with Ad5-prM-E or Ad4-prM-E at either 500 vp/cell or an MOI of 1. They were incubated at 37 °C and 5% CO_2_ and harvest at 48 hours. Cells were denatured using Laemmli buffer plus 2-mercaptoethanol and boiled at 100 °C for 10 minutes. The sample was then passed through a QIAshredder (Qiagen). Sample was loaded onto a 12.5% SDS-PAGE gel and separated using electrophoresis. Protein was transferred to a nitrocellulose membrane and blocked for 30 minutes with 5% milk in TBST. The membrane was incubated in mouse anti-ZIKV E protein antibody (mAb-0302156; BioFront Technologies) at 1:5000 and mouse anti-GAPDH (sc-47724; Santa Cruz Biotechnology, Inc.) at 1:2000 in TBST 1% milk overnight at 4 °C. After 3 washes in TBST, the membrane was incubated with goat anti-mouse-HRP conjugated antibody (Millipore Sigma) at 1:2000 in TBST 1% milk for 1 hour at room temperature. After incubation, the membrane was washed and developed with SuperSignal West Pico Chemiluminescent Substrate (Thermo Scientific).

### ZIKV plaque assay

Cell supernatants were serially diluted in DMEM 2% FBS and added to confluent Vero cells. After incubation with rocking at 37 °C and 5% CO_2_ for 1 h, cells were washed once with PBS and 1 mL of agar/DMEM overlay was added. Plates were incubated for 4 days at 37 °C and 5% CO_2._ After 4 days, cells were fixed with 10% formaldehyde, the agar plugs removed, and the cell monolayer was stained using crystal violet. Plaques were counted and plaque forming unit (PFU) per mL were calculated.

### ZIKV foci forming unit assay

Virus stocks were titrated by focus forming assay (FFA) on Vero cells as previously described^39^ and stored in aliquots at −80 °C.

### Mouse challenge studies

Female *Ifnar1*^*−*/*−*^ mice (8-9 weeks old) were immunized intramuscularly at day 0 with 10^10^ vp of Ad4-prM-E (n = 9), Ad5-prM-E (n = 10), or control PBS (n = 10). Two experimental replicates were performed however, after day 4, three mice in the Ad4-prM-E group were lost to unrelated causes due to anesthesia complications. Female C57BL/6 mice were immunized intramuscularly at day 0 with 10^10^ vp of the indicated Ad vaccine or control PBS. All mice were boosted at week 3 with 10^10^ vp of indicated Adenovirus or sham vaccine and then challenged at week 7 with 10^6^ FFU of mouse-adapted ZIKV-Dakar 41525 via the intraperitoneal (i.p.) route. C57BL/6 mice were administered 2 mg of anti-mouse Ifnar1 mAb (MAR1-5A3, BioXcell) via the i.p. route one day before ZIKV infection and given 0.5 mg more at day 4 post infection. All mice were monitored daily for weight loss and mice were euthanized at 25% weight loss.

### Antibody and T-cell assays

Female C57BL/6 mice were immunized with 10^10^ vp of indicated Adenovirus or control PBS. All immunizations were performed intramuscularly with a 27-gauge needle into both quadriceps in two 25 µl injections. At week 6, prime/boost animals were boosted with 10^10^ vp of indicated Adenovirus. At week 8, all animals were terminally bled via a cardiac puncture and spleens were harvested. Sera was isolated from whole blood with a BD Microtainer Blood Collection Tube (Becton Dickinson) and used for further ELISA and neutralization tests. Splenocytes were harvested for T cell immune assays.

### ELISA

Immunolon 4 HBX microtiter 96-well strips (VWR) were coated with 150 ng of recombinant ZIKV E protein (Cat #:MBS596088; MyBioSource) in bicarbonate/carbonate coating buffer overnight at 4 °C. The plates were blocked with 2.0% BSA in PBS for 2 hours at room temperature (RT). Sera was diluted in 1.0% BSA in PBS and incubated for 2 hours at RT. The plates were washed 6X with PBST and incubated with goat anti-mouse-HRP antibody (1:2000; Thermo Fisher) in 1.0% BSA in PBS for 1 hour at RT. After washing 4X with PBST and 2X with PBS, the plate was developed with 1-Step Ultra TMB-ELISA (Thermo Fisher), and the reaction was stopped with 2 M sulfuric acid. The OD450 was detected using a SpectraMax i3x Multi-Mode microplate reader (Molecular Devices), and the endpoint titer was determined as signal that was two times background values.

### ELISPOT

The T-cell epitopes were mapped using a peptide array of the ZIKV strain PRVABC59 E protein from BEI resources (Catalog No. NR-50553). This 164-peptide array spans the entire E region and consists of 15-mers with 12 amino acid overlap. Potential immunogenic peptides were identified using a matrix of peptides pools, and the epitopes were confirmed using individual peptides. Splenocytes were isolated from mice using a 40 μm Nylon cell strainer (BD Labware). Red blood cells were lysed using ACK lysis buffer and the splenocytes were resuspended in cRPMI at a concentration of 10^6^ splenocytes/mL. Ninety-six well polyvinylidene difluoride-backed plates (MultiScreen-IP, Millipore) coated with 50 μl of anti-mouse IFN-γ mAb AN18 (5 µg/ml; Mabtech) overnight at 4 °C. Plates were washed and blocked with RPMI at 37 °C for 1 hour. Equal volumes (50 µL) of the single-cell suspension splenocytes and peptide (5ug/mL) were added to the wells in duplicate. Plates were incubated overnight at 37 °C with 5% CO_2_. The plates were washed 6X with PBS and incubated with 100 μl of biotinylated anti-mouse IFN-γ mAb (1:1000 dilution; Mabtech) diluted in PBS with 1.0% FBS for 1 hour at RT. Plates were washed 6X with PBS and incubated with 100 µl of streptavidin-alkaline phosphatase conjugate (1:1000 dilution; Mabtech) diluted in PBS 1.0% FBS. After 1 hour at RT, the plates were washed 6x with PBS. To develop, 100 µl of BCIP/NBT (Plus) alkaline phosphatase substrate (Thermo Fisher) was added to each well and development was stopped by washing several times in dH_2_O. The plates were air dried and spots were counted using an automated ELISpot plate reader (AID iSpot Reader Spectrum). Results are expressed as spot-forming cells (SFC) per 10^6^ splenocytes.

### qPCR for viral load quantification

Challenged mice were bled from the submandibular vein at day 4 post infection and sera was isolated. RNA from sera was extracted using the PureLink Viral RNA/DNA Mini Kit according to manufacturer’s instructions (Invitrogen). Real time RT-qPCR was performed using the Luna Universal Probe One-Step RT-qPCR Kit (NEB). It was run on a QuantStudio 3 Real-Time PCR System (Applied Biosystems) using the Luna kit cycling conditions (55 °C for 10 min, 95 °C for 1 min, and 40 cycles of 95 °C for 10 s and 60 °C for 1 min). Results were compared to a standard curve created using ZIKV RNA extracted from a known quantity of infectious virus. Unamplified samples were set at the limit of detection (100 FFU equivalent/mL). The following primer probe set was used: 1183 F: 50-CCACCAATGTTCTCTTGCAGACATATTG-30; 1268 R: 50-TTCGGACAGCCGTTGTCCAACACAAG-30; and probes (1213 F): 5′FAM/AGCCTACCTTGACAAGCAGTC/BHQ1–3′^22^.

### Plaque reduction neutralization assay

Mouse sera was heat-inactivated at 56 °C for 30 min. Sera then was diluted 2-fold in DMEM 2% FBS and incubated with 50 PFU of ZIKV strain PRVABC59 for 2 hours at 37 °C and 5% CO_2._ This was then added to a 24-well plate of confluent Vero cells. The plaque assay was continued as described above.

### Statistical analysis

GraphPad Prism software was used to analyze all data. Data are expressed as the mean with standard error (SEM). Survival curves were analyzed using the log rank test and weight loss was analyzed using two-way ANOVA multiple comparison test. ELISA, PRNT50, T-cell data, and viral burden were analyzed using one-way ANOVA with Bonferroni multiple comparisons. A p value < 0.05 was considered statistically significant (^*^p < 0.05; ^**^p < 0.01; ^***^p < 0.001; ^****^p < 0.0001).

## Electronic supplementary material

Supplemental Info
