# Supplementary material for: Efficacy of a T Cell-Biased Adenovirus Vector as a Zika Virus Vaccine
Source: Sci Rep. 2018 Dec 20;8:18017. doi: 10.1038/s41598-018-35755-z (PMC6301965; doi:10.1038/s41598-018-35755-z)
Supplement: Supplementary file 1 — Supplemental Info [file 41598_2018_35755_MOESM1_ESM.pdf]

Supplemental Data

# Efficacy of a T Cell-Biased Adenovirus Vector as a Zika Virus Vaccine

Brianna L. Bullard[1], Brigette N. Corder[1], Matthew J. Gorman[2], Michael S. Diamond[2], and Eric A. Weaver[1*]

Supplementary Figure 1

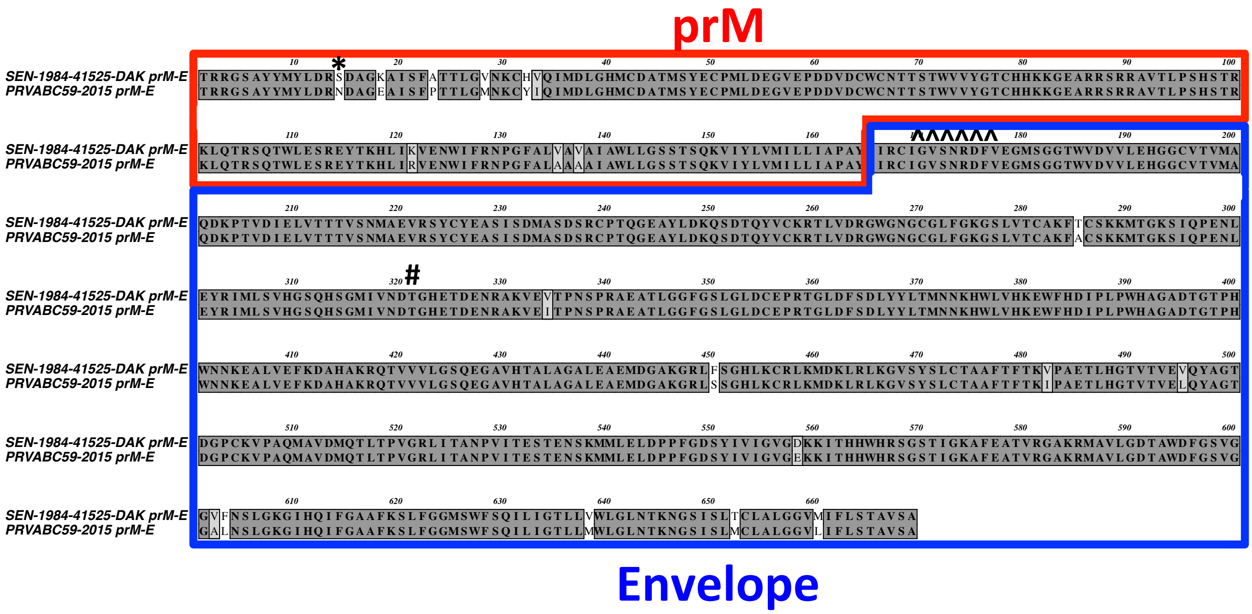


**Supplementary Figure 1**

**Alignment of the African strain ZIKV Dakar 41525 to the Asian strain ZIKV PRVABC59.**

A ClustalW alignment of the prM-E region from the African ZIKV Dakar 41525 strain used in the challenge studies and the Asian ZIKV PRVABC59 strain used in the construction of the vectored vaccine. The CD8$^+$ T cell epitope $E_{4-12}$ is indicated by a ^ symbol(37). The * symbol indicates a S139N mutation that is associated with increased virulence in neural progenitor cells (54). The # symbol indicates a motif associate with increased virulence (55).

# Supplementary Table 1

| Peptide # | Sequence | Position (E#1-504) |
|---|---|---|
| 1 | IRCIGVSNRDFVEGM | 1-15 |
| 2 | IGVSNRDFVEGMSGG | 4-18 |
| 3 | SNRDFVEGMSGGTWV | 7-21 |
| 6 | SGGTWVDVVLEHGGC | 16-30 |
| 8 | DVVLEHGGCVTVMAQ | 22-36 |
| 19 | EVRSYCYEASISDMA | 55-69 |
| 21 | YEASISDMASDSRCP | 61-75 |
| 24 | SDSRCPTQGEAYLDK | 70-84 |
| 25 | RCPTQGEAYLDKQSD | 73-87 |
| 102 | SYSLCTAAFTFTKIP | 304-318 |
| 103 | LCTAAFTFTKIPAET | 307-321 |
| 104 | AAFTFTKIPAETLHG | 310-324 |
| 119 | VGRLITANPVITEST | 355-369 |
| 120 | LITANPVITESTENS | 358-372 |
| 123 | ESTENSKMMLELDPP | 367-381 |
| 124 | ENSKMMLELDPPFGD | 370-384 |

## Supplementary Table 1

**The positive peptides in T-cell mapping of the E region of ZIKV.**

The peptides determined to be positive during the T-cell mapping of the Envelope region of ZIKV as indicated in Fig. 3. Peptides were positive if the response was 4 times that of the PBS sham vaccinated mice. Amino acid sequence and position number on the E protein are indicated.

Supplementary Figure 2

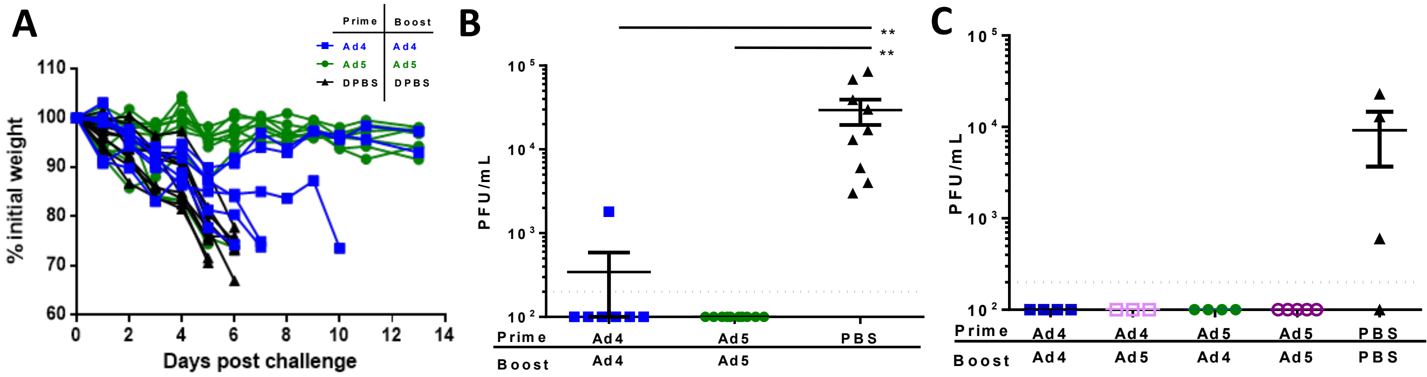


## Supplementary Figure 2

**Individual mice weight loss during the Ifnar1$^{-/-}$ mice challenge and viremia on day 4 post infection based on plaque assay.**

The individual mouse weights during the challenge of the *Ifnar1*$^{-/-}$ mice in Fig. 5 are plotted in (A). The plaque forming units per mL (PFU/mL) in the sera at day 4 post infection in the *Ifnar1*$^{-/-}$ challenge model (B) ($p<0.01$; one-way ANOVA) and anti-Ifnar1 challenge model (C). Data are expressed as the mean with standard error (SEM).

500 vp/cell | 1 MOI

Ad5-prME | Ad4-prME | Ad5-prME | Ad4-prME | Uninfected

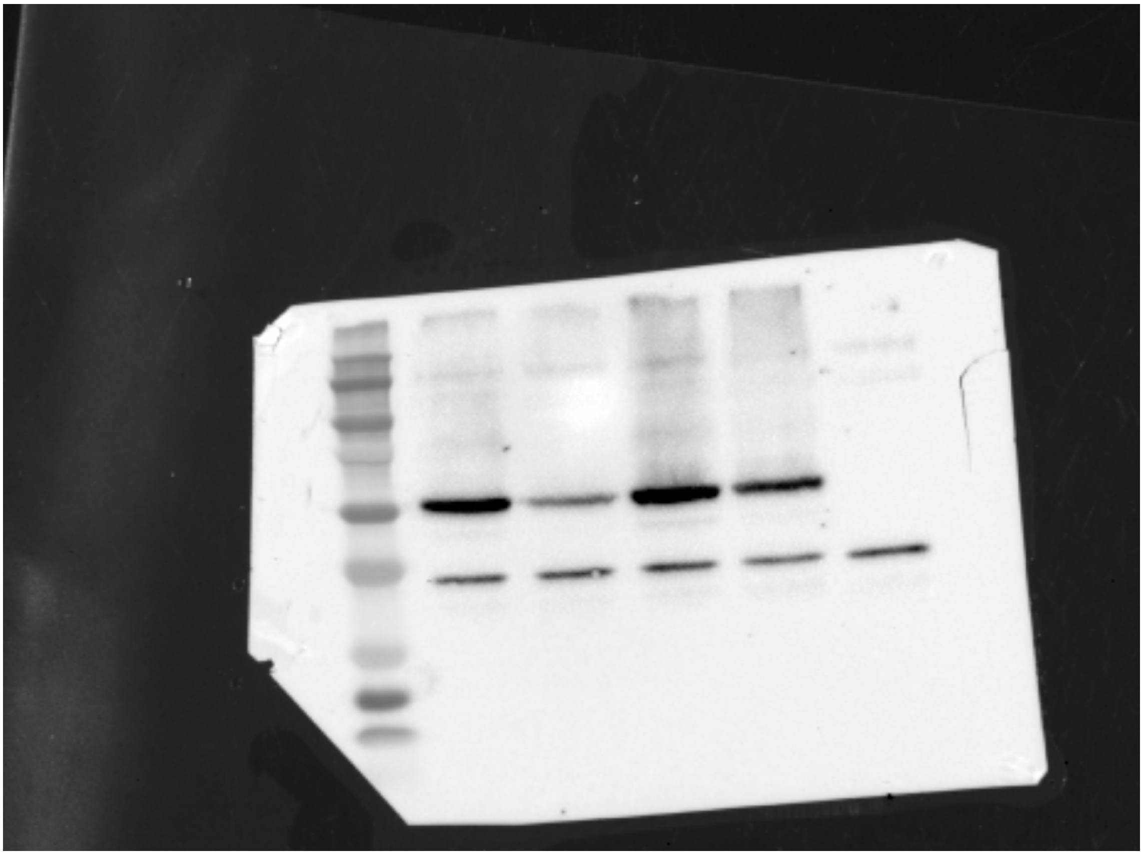

ZIKV-E

GAPDH
